# Weighted Single-Step GWAS Identifies Genes Influencing Fillet Color in Rainbow Trout

**DOI:** 10.3390/genes13081331

**Published:** 2022-07-26

**Authors:** Ridwan O. Ahmed, Ali Ali, Rafet Al-Tobasei, Tim Leeds, Brett Kenney, Mohamed Salem

**Affiliations:** 1 Department of Animal and Avian Sciences, University of Maryland, College Park, MD 20742, USA; rahmed20@umd.edu (R.O.A.); areali@umd.edu (A.A.); 2 Computational Science Program, Middle Tennessee State University, Murfreesboro, TN 37132, USA; rafet.al-tobasei@mtsu.edu; 3 United States Department of Agriculture Kearneysville, National Center for Cool and Cold Water Aquaculture, Agricultural Research Service, Kearneysville, WV 25430, USA; tim.leeds@usda.gov; 4 Division of Animal and Nutritional Sciences, West Virginia University, Morgantown, WV 26506, USA; bkenney@wvu.edu

**Keywords:** fillet color, rainbow trout, GWAS, genetic markers, genes

## Abstract

The visual appearance of the fish fillet is a significant determinant of consumers’ purchase decisions. Depending on the rainbow trout diet, a uniform bright white or reddish/pink fillet color is desirable. Factors affecting fillet color are complex, ranging from the ability of live fish to accumulate carotenoids in the muscle to preharvest environmental conditions, early postmortem muscle metabolism, and storage conditions. Identifying genetic markers of fillet color is a desirable goal but a challenging task for the aquaculture industry. This study used weighted, single-step GWAS to explore the genetic basis of fillet color variation in rainbow trout. We identified several SNP windows explaining up to 3.5%, 2.5%, and 1.6% of the additive genetic variance for fillet redness, yellowness, and whiteness, respectively. SNPs are located within genes implicated in carotenoid metabolism (β,β-carotene 15,15′-dioxygenase, retinol dehydrogenase) and myoglobin homeostasis (ATP synthase subunit β, mitochondrial (*ATP5F1B*)). These genes are involved in processes that influence muscle pigmentation and postmortem flesh coloration. Other identified genes are involved in the maintenance of muscle structural integrity (kelch protein 41b (*klh41b*), collagen α-1(XXVIII) chain (*COL28A1*), and cathepsin K (*CTSK*)) and protection against lipid oxidation (peroxiredoxin, superoxide dismutase 2 (*SOD2*), sestrin-1, Ubiquitin carboxyl-terminal hydrolase-10 (*USP10*)). A-to-G single-nucleotide polymorphism in β,β-carotene 15,15′-dioxygenase, and *USP10* result in isoleucine-to-valine and proline-to-leucine non-synonymous amino acid substitutions, respectively. Our observation confirms that fillet color is a complex trait regulated by many genes involved in carotenoid metabolism, myoglobin homeostasis, protection against lipid oxidation, and maintenance of muscle structural integrity. The significant SNPs identified in this study could be prioritized via genomic selection in breeding programs to improve fillet color in rainbow trout.

## 1. Introduction

The aquaculture industry produces food fish to satisfy a growing US and worldwide demand. Rainbow trout is the most cultivated, cool, freshwater fish in the United States [1]. Aquaculture supplies protein with low saturated fat and cholesterol content and high omega-3 fatty acids [2,3]. Rainbow trout are reared to produce fillets, and high production efficiency is needed to meet the ever-increasing demand for quality products. A significant constraint is the lack of genetically improved fish strains with high fillet yields and good-quality fillets. The industry has worked to remedy the situation by introducing breeding programs to select the best animals as parents for the next generations. Many of these breeding programs are traditional, using phenotypic information from breeding candidates and their pedigree to make selection decisions [4]. However, traditional breeding programs can be time-consuming and inefficient, especially for lethal traits such as fillet yield and color that cannot be accurately measured on live fish [5].

Use of genomic information in breeding programs offers a faster and more accurate method of achieving genetic progress. Achieving this goal requires understanding the genetic architecture underlying the variability in these traits. Genome-wide association (GWA) studies can identify genome regions associated with desired traits. GWA studies take advantage of linkage disequilibrium between SNP markers and genetic loci controlling a trait of interest. GWA studies have been conducted in the rainbow trout breeding program at the National Center for Cool- and Cold-Water Aquaculture (NCCCWA) for growth [6], muscle yield [7], intramuscular fat [8], fillet firmness [9], and disease resistance [10]. Another essential trait that requires attention is the fillet color—targeted in this study.

Fillet color is an important quality trait, usually influencing consumers’ satisfaction and point-of-purchase decisions. There are two markets for rainbow trout fillets—one for a bright reddish/pink fillet and one for bright white fillets. Consumers usually reject or downgrade pale yellowish fillets. Factors affecting fillet color range from genetics to environmental factors and to harvest, handling, and storage conditions [11]. Postmortem fillet color stability also depends on the rate of myoglobin oxidation, which is influenced by oxidation of intramuscular lipids and mitochondrial activity [12]. Salmonids’ characteristic pink/bright reddish fillet color results from the deposition of naturally occurring carotenoids or synthetic pigments added to the diets [13]. Carotenoids supplied in the diet are transported through the intestinal wall, metabolized within the cells of the intestinal linings or in the liver. The unmetabolized portion is deposited in the muscle by binding to muscle α-actin [14,15]. Rainbow trout flesh will typically be less reddish or whitish when the diet is not supplemented with carotenoids, as salmonids cannot synthesize carotenoids de novo [16]. Atlantic salmon and rainbow trout fish fed an unpigmented diet yield fillets with higher L* (lightness) and lower a* (redness) and b* (yellowness) values in comparison to fish on astaxanthin-supplemented diets [17,18]. Brown et al. [19] reported a significant difference in the color retention indices (ECI, hue, and chroma) of fillets from rainbow trout fish that never received dietary astaxanthin compared to fillets from fish that received an astaxanthin-supplemented diet. Even when fed a non-pigmented diet, Crouse et al. [20] observed significant differences in fillet redness (a*) between different rainbow trout strains fed the same diet. Red/pink pigmented rainbow trout fillets are deemed more desirable and marketed at a higher price than white fillets [19,21,22], but some consumers, especially in the US, may prefer a whiter fish.

Astaxanthin, a carotenoid added to the salmonid feed to improve the reddish color of the fillet, is an expensive feed ingredient, accounting for up to 30% of the feed cost. Therefore, development of genetically improved rainbow trout strains that more efficiently incorporate carotenoids into the muscle will benefit the aquaculture industry by improving profitability and consumer satisfaction. When fed unpigmented diets, genetically improved strains will also use naturally occurring carotenoids in the feed ingredients.

Studies in humans [21,22], chicken [23], mice [24], and Atlantic salmon [25] identified the β-carotene 15,15′-oxygenase (BCO1) enzyme as responsible for variation in the ability to metabolize carotenoids. Additionally, a recent study on a rainbow trout line used for commercial production in France identified Bcmo1 (β,β-carotene 15,15-dioxygenase), dkk3a (dickkopf WNT signaling pathway inhibitor 3a), and bola3 (bolA family member 3) as possible genes whose functions regulate the color of rainbow trout fillets [26]. Sae-Lim et al. [27] used the multi-trait GWAS approach to account for the relationship between body weight and fillet color and identified BCO1 and ppa1b (inorganic pyrophosphatase) within QTL regions influencing fillet color in Atlantic salmon. Other studies identified ATP-binding cassette subfamily G member 2 (abcg2-1a) in Atlantic salmon [28], *PyBCO-1* in Scallop [29], and BCO2 in Chinook salmon [30] as candidate genes for fillet color. However, there is still much to learn about the genetic architecture of fillet color before it can be incorporated into breeding programs through genomic selection.

This study aims to use GWA analysis to identify genomic regions associated with fillet color traits (redness, yellowness, lightness, and whiteness) in a population of rainbow trout developed at the NCCCWA that had undergone five generations of selection for growth rate. Fish were fed an unpigmented commercial fishmeal-based diet.

## 2. Materials and Methods

### 2.1. Fish Population and Phenotype Used for GWA in This Study

The rainbow trout fish population used in this study was from a growth-selected line from NCCCWA, as described by Leeds et al. [31]. Fish from the third (hatch-year 2010) and fourth (hatch-year 2012) generations belonging to 197 families were included in this study. The breeding, selection, feeding, rearing, and harvesting procedures are as described by Salem et al. [7]. The fish used in this study were fed an unpigmented commercial fishmeal-based diet (42% protein, 16% fat; Ziegler Bros Inc., Gardners, PA, USA) using automatic feeders (Arvotec, Huutokoski, Finland). Initially, young fish were fed at a daily rate of ∼2.5% of body weight (BW), gradually reduced to approximately 0.75% of BW.

Fillet color parameters, L*, a*, and b*, which represent lightness, redness, and yellowness, respectively, were obtained from the fresh fillet surface using the Minolta Chroma Meter CR-200 (Minolta, Model CR-300; Minolta Camera Co., Osaka, Japan). The parameters were recorded a day after harvest at three locations above the lateral line of the right-side fillet, as described by Al-Tobasei et al. [32]. In addition to the standard color parameters, L*, a*, and b*, the fillet whiteness index was calculated using the equation: Whiteness = 100 − [(100 − L*)^2^ + a*^2^ + b*^2^]^1/2^ [33]. The data were obtained from 878 fish from 2 harvest years: 406 from hatch-year 2010 and 472 from hatch-year 2012.

### 2.2. Genotyping and Quality Control

The 878 fish were genotyped with the 50k transcribed SNP-chip developed and described before [7]. PREGSF90 [34] was used to perform quality control using the following criteria: call rate for SNP and samples > 0.90, MAF > 0.05, monomorphic = 1, and HWE < 0.15. In total, 32,868 SNPs passed the QC and were used for subsequent analysis.

### 2.3. Descriptive Statistics

The mean and standard deviation values of each fillet color phenotype were calculated. Heritability was estimated as the ratio of additive genetic variance to total phenotypic variance. Variance components were estimated using the restricted maximum likelihood method found in AIREML in BLUPF90 software [34] using the following linear mixed model:

y = Xb + Z_1_a + Z_2_w + *e*

where y is the vector of phenotypes, b is the vector of fixed effects (age, harvest group, and hatch-year), a is the vector of additive genetic effect, w is the vector of random family effect, and *e* is the residual effect. X, Z_1_, and Z_2_ are incidence matrices for the effects contained in b, a, and w, respectively.

### 2.4. Genome-Wide Association Analysis

The weighted single-step GBLUP (wssGBLUP) approach proposed by Wang et al. [35] was used to perform genome-wide association analysis using the BLUPF90 family programs [34]. This method allows the use of genotyped and ungenotyped animals while integrating phenotype, genotype, and pedigree information in a mixed model for single-trait analysis.

The four fillet color parameters (L*, a*, b*, and whiteness) were analyzed using the single-trait animal model in wssGBLUP according to the model below:

y = Xb + Z_1_a + Z_2_w + *e*

where y is the vector of phenotypes, b is the vector of fixed effects, a is the vector of additive genetic effect, w is the vector of random family effect, and *e* is the residual effect. X, Z_1_, and Z_2_ are incidence matrices for the effects contained in b, a, and w, respectively. The fixed effects used in this study are fish age, harvest group, and hatch-year. The assumptions are that a~N(0, H$\sigma_{a}^{2}$) and e~N(0, *I*$\sigma_{e}^{2}$), where $\sigma_{a}^{2}$ and $\sigma_{e}^{2}$ are the additive genetic variance and residual variance, respectively. The H is a blend of pedigree and SNP-derived matrix [36], while *I* denotes the Identity matrix. The inverse of H is used in the wssGBLUP mixed model analysis [37].

$$H^{-1}=A^{-1}+\left[\begin{array}{cc} 0 & 0\\ 0 & G^{-1}A_{22}^{-1} \end{array}\right]$$

where *A*^−1^ is the inverse of the pedigree relationship matrix for all animals, $A_{22}^{-1}\;$is the inverse of the pedigree relationship matrix of genotyped animals, and *G*^−1^ is the inverse of the genomic relationship matrix. The random family effect is uncorrelated and only accounts for the fact that the animals within the same family were raised in a common environment, and the covariance structure is given by *I*$\sigma_{w}^{2}$, where *I* is an identity matrix and $\sigma_{w}^{2}$ is the family variance.

AIREMLF90 was used to estimate variance components supplied to BLUPF90 to predict genomic estimated breeding values (GEBV). The inbreeding coefficient was calculated from the pedigree data of 1420 fish by RENUMF90 using the method of Meuwissen and Luo [38].

BLUPF90 was used to predict breeding values using a weighted genomic relationship matrix (G). The SNP marker effect and new weights were then computed with POSTGSF90 [34] using 50 adjacent SNP sliding windows. All SNPs were initially assumed to be equally weighted (i.e., given an equal weight of 1.0). The final SNP weights and SNP effects were estimated using the option “non-linear A”, which allows for stable SNP weights after some iterations. Non-linear prediction assumes prior non-normal distribution of the marker effect and that markers do not contribute equally to genetic variance [39]. The non-linear approach resulted in greater reliability in the genomic prediction breeding value for bulls [39].

The percentage of additive genetic variance explained by each SNP window was calculated as:

$$\frac{var\left(a_{i}\right)}{\sigma_{a}^{2}}\times 100\%=\frac{var\left(\Sigma_{j=i}^{50}Z_{j}\mu_{j}\right)}{\sigma_{a}^{2}}\times 100\%$$

where *a_i_* is the genetic value of the *i*-th window consisting of 50 adjacent SNPs, *σ*^2^ is the total genetic variance, *z_j_* is a vector genotype of the *j*-th SNP for all animals, and $\mu_{j}$ is the SNP effect of the *j*-th SNP within the *i*-th window.

The qqman package [40] was used to obtain Manhattan plots for the proportion of additive genetic variance explained by each SNP window.

### 2.5. Identification of Candidate Genes

Genomic windows explaining at least 1% of the genetic variance were selected as possible genetic regions associated with the fillet color traits. The 1% threshold was set based on the literature obtained [41,42,43]. The SNPs were annotated using the NCBI rainbow trout genome assembly (GCF_013265735.2) to identify SNP-harboring genes. We used a literature search to identify relevant gene pathways and functions to understand the possible mechanisms by which the candidate genes regulate the traits. Genes previously identified in the literature or found to be related to color traits were further discussed.

### 2.6. MicroRNA Target Prediction

SNPs located in the 3’UTR of genes associated with fillet color in this study were investigated if their 3’UTR served as a target site for rainbow trout microRNAs. MicroRNA targets were predicted using three algorithms (*PITA*, *miRanda*, and *TargetSpy*) from the sRNAToolbox (http://bioinfo5.ugr.es/srnatoolbox). The rainbow trout microRNA repertoire was obtained from Juanchich et al. [44].

## 3. Results

### 3.1. Descriptive Statistics and Heritability Estimates for the Color Traits

There was more variation in the redness (54%) and yellowness (30%) in comparison to lightness and whiteness (6%) (Table 1). The heritability estimates of the traits in this population were moderate (0.16–0.39). The phenotypic correlation between lightness (L*) and whiteness color indices was 0.99 (R^2^ = 0.98), whiteness and redness (a*) was 0.28, whiteness and yellowness (b*) was 0.67, and redness (a*) and yellowness (b*) was 0.45 (Table 1).

### 3.2. Genome-Wide Association Study and QTL Identification

A weighted single-step GBLUP approach was implemented in the BLUPF90 family of programs [34] to identify SNPs associated with fillet color traits. The GWAS results for whiteness and lightness color indices follow the same pattern, as expected, because of the high phenotypic correlation. Subsequently, only the whiteness trait will be discussed further. We identified 244, 161, and 115 SNPs in genomic windows, explaining at least 1% of the genetic variation in fillet redness, yellowness, and whiteness, respectively (Table 2 and Table 3, and Supplementary Table S1). The SNPs were identified within a genomic sliding window of 50 SNPs.

**Figure 1:**
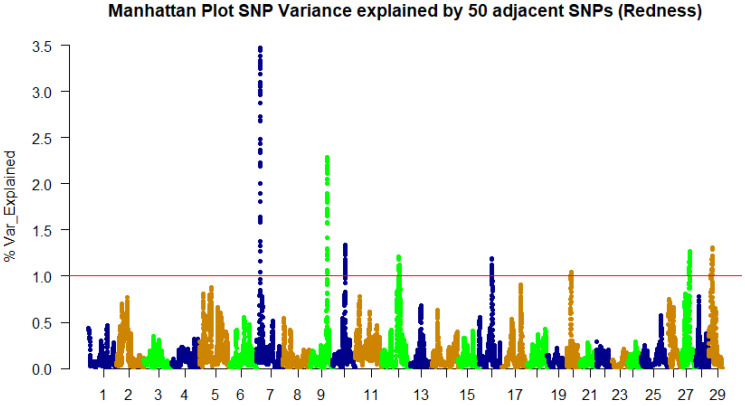
Manhattan plot of percent of genetic variance explained by 50 adjacent SNP windows for fillet redness (a*).

For redness (a*), chromosome 7 harbors the majority (33%) of the SNPs (80), followed by chromosome 9 (67 SNPs) (Figure 1, Table 2). Forty-five percent of the SNPs are in untranslated regions of genes, forty-two percent are in the coding regions. The highest peak corresponds to a SNP window on chromosome 7 that explains ~3.5% of the genetic variance.

**Figure 2:**
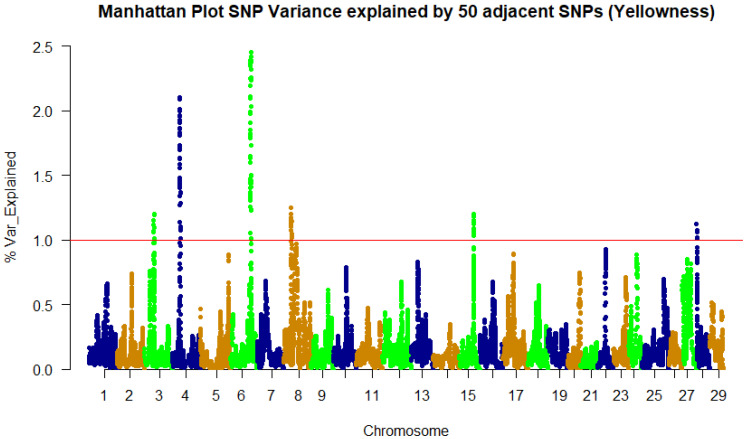
Manhattan plot of percent of genetic variance explained by 50 adjacent SNP windows for fillet yellowness (b*).

For the yellowness trait (b*), most of the SNPs (66) are resident in chromosome 6 (41%), followed by 46 SNPs on chromosome 4 (29%). The peak SNP window, resident on chromosome 6, explains up to ~2.5% of the genetic variance for this trait (Figure 2, Table 2). Forty percent of the SNPs are in untranslated regions (UTR), while forty-seven percent are in coding regions.

**Figure 3:**
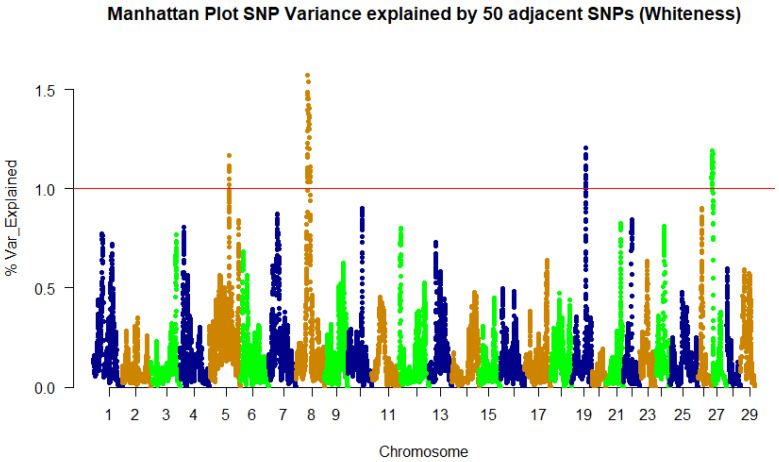
Manhattan plot of percent of genetic variance explained by 50 adjacent SNP windows for the fillet whiteness.

Lightness (L*) and whiteness are similar in their genetic architecture, with peak SNP on chromosome 8 explaining only 1.6% of the genetic variance for this trait (Figure 3, Table 3). Forty-three percent of the SNPs are found within gene-coding regions, while forty-five percent are located in untranslated regions.

### 3.3. MicroRNA Target Prediction

Our results revealed that the 3’UTR region of *ANKH* (ANKH inorganic pyrophosphate transport regulator), *RETRIG1* (reticulophagy regulator 1), and *HSPB1* (heat-shock protein, α-crystallin-related, 1) genes are target sites for the omy-mir-1388-3p, omy-mir-219-5p, and omy-miR-724-5p microRNAs, respectively. An A-to-T single-nucleotide substitution at the target site of omy-mir-1388-3p causes a loss of its miRNA target site. Likewise, a C-to-T transition at the 3’UTR of *HSPB1* resulted in a loss of the target site for the omy-miR-724-5p miRNA. Single-nucleotide substitution at the target site of omy-mir-219-5p does not lead to a loss of the target site.

## 4. Discussion

Fillet color is an important quality trait in salmonids influencing consumers’ purchasing decisions. Therefore, the industry is interested in selecting rainbow trout with superior genetic merit in their ability to produce a bright red or white fillet. Understanding the trait’s genetic architecture is required to determine the best genetic improvement approach. In this study, a genome-wide association investigation identifies regions of the genome influencing variability in fillet color traits in rainbow trout.

### 4.1. Descriptive Statistics and Heritability Estimates for the Color Traits

There is more variation in redness (a*) and yellowness (b*) compared to whiteness. The estimated heritability for fish in this population is low to moderate, similar to an estimate of 0.27 obtained for a rainbow trout fillet color score by Gjerde and Schaeffer [45]. A heritability estimate of 0.30 was recorded for fillet redness by Haffray et al. [46]. Blay et al. [26] reported higher heritability estimates of 0.46, 0.45, and 0.28 for rainbow trout fillet lightness (L*), redness (a*), and yellowness (b*), respectively. Overall, these studies demonstrate the possibility of achieving genetic improvement for fillet color traits through selection.

### 4.2. Summary of wssGWAS for Fillet Color Traits

The SNP windows explaining the highest genetic variance are found on chromosomes 7, 6, and 8 for fillet redness, yellowness, and whiteness, respectively. The SNP-harboring genes were classified according to their function and relevance to fillet color into the following categories.

### 4.3. Genes Involved in Carotenoid Metabolism

β, betacarotene 15,15-dioxygenase and retinol dehydrogenase are involved in carotenoid metabolism [47,48,49]. Fish species such as Atlantic salmon and rainbow trout deposit carotenoids in their muscle that enhance the reddish coloration of the fillet [15] and variation in carotenoid metabolism is associated with β-carotene oxygenase-1 function. Similar to the findings of this study, β, betacarotene 15,15-dioxygenase was implicated in its association with rainbow trout fillet yellowness [26]. Helgeland et al. [25] identified β-carotene oxygenase-1 (*BCO1*) and its paralogue β-carotene oxygenase-1 (*BCO1L*) as two probable causal genes influencing flesh color in Atlantic salmon. They supported their findings with functional studies of mRNA and protein expression, which pointed to *BCO1L* as the most likely of the two genes to influence flesh color variation. Several studies have identified single-nucleotide polymorphism within *BCO1* that was associated with breast meat color in chicken [25,48,49]. In the mollusk, Yesso scallop, GWAS, and gene expression studies were used to confirm that PyBCO (a homolog of *BCO1* in fish) was responsible for carotenoid metabolism and subsequent muscle coloration [29].

β,β-carotene 15,15′-dioxygenase on chromosome 6 explains 2.2% of the phenotypic variance for yellowness, while retinol dehydrogenase-7 found on chromosome 7 explains 2.9% of the variation in the redness trait (Table 2). A-G SNP in β,β-carotene 15,15′-dioxygenase causes isoleucine-to-valine non-synonymous amino acid substitution. Transcriptome analysis identifies retinol dehydrogenase-12 as a candidate gene regulating body-color formation in ornamental shrimp [50]. Carotenoids can serve as exogenous antioxidants to prevent cell oxidative damage, and these pigments inhibit lipid peroxidation and hemoglobin oxidation in human erythrocytes [51].

### 4.4. Genes Involved in Myoglobin Homeostasis and Protection against Lipid Oxidation

*ATP5F1B,* methylmalonyl-CoA mutase, *ABC11*, calsequestrin, cytochrome b5 (*CYB5*), ubiquitin carboxyl-terminal hydrolase 10 (*USP10*), peroxiredoxin, superoxide dismutase 2 (*SOD2*), sestrin-1, myosin X, and protein PRRC2C are genes that were found to affect fillet color in the study (Table 2 and Table 3). They are known to play a role in either myoglobin homeostasis or regulation of lipid peroxidation.

ATP synthase subunit β, mitochondrial (*ATP5F1B*) on chromosome 7 is another identified gene explaining over 3.5% of the genetic variability of fillet redness in rainbow trout (Table 2). It generates ATP from ADP through the electron transport system of the respiratory chain in the mitochondria [52]. Myoglobin is a muscle protein that binds oxygen and is responsible for muscle coloration [53]. Myoglobin exists in three forms: deoxymyoglobin, oxymyoglobin, and metmyoglobin. Although with low concentration of heme in the muscle, studies in salmonids have indicated that flesh color is, to some extent, dependent on the status of myoglobin [54,55]. The oxymyoglobin form promotes bright-reddish coloration in beef and salmon fillets, while metmyoglobin promotes fillet lightness (L*) [53,54]. The mitochondria function influences conversion between the three myoglobin forms [56]. Mitochondrial function can remain in postmortem muscle, influencing the conversion between myoglobin forms and the meat’s color [56,57]. Ramanathan et al. [53] suggested that understanding factors that influence mitochondrial function is key to unraveling the regulation of beef color appearance. Similarly, the gene *ATP5F1B* may influence rainbow trout fillet color by regulating mitochondrial integrity and function. Methylmalonyl-CoA mutase regulates mitochondria function by catalyzing the isomerization of methylmalonyl-CoA to succinyl-CoA [58]. It explains 1.29% of the genetic variability for fillet whiteness (Table 3).

Bile salt export pump *(ABCB11*) on chromosome 7 explains ~2.9% of the genetic variation in fillet redness (Table 2). This gene participates in bile acid homeostasis in an ATP-dependent manner [59,60]. It affects lipid metabolism and oxidation by regulating biliary tract lipid acid secretion through its action on bile salts’ excretion [61,62,63]. The influence of lipid oxidation on myoglobin, and thus meat color, is essential in meat color research. Postmortem meat color stability is affected by the muscle’s lipid oxidation rate [12]. The lipid auto-oxidation process generates free radicals and secondary products such as aldehydes and ketones that accelerate myoglobin oxidation [12] and, consequently, meat color deterioration [64,65,66]. Lipid peroxidation in the bile may generate pro-inflammatory agents by converting free fatty acids into lipid peroxides and aldehydes [67,68]. Chen et al. [66] discovered that aldehydes, a lipid oxidation product, accelerate the rate of myoglobin oxidation and promote permeability of the mitochondrial membrane. This process inhibits electron-transport chain-mediated metmyoglobin reduction and could profoundly affect fillet color stability, as discussed above with the ATP synthase subunit β, mitochondrial (*ATP5F1B*) gene. Blay et al. [26] identified two genes, dkk3 and bola3, known to be involved in adipogenesis, as genes harboring regulatory regions associated with fillet color. This work supports a relationship between fillet color and intramuscular fat content.

Conversion between the three myoglobin forms is influenced by mitochondrial function [56]. Cytochrome B5, a metmyoglobin reductase, reduces ferric myoglobin (methemoglobin) to ferrous myoglobin within muscle mitochondria [69,70]. In this study, cytochrome b5 (*CYB5*), on chromosome 6, explained up to 2.3% of the genetic variability for fillet yellowness (Table 2). This gene may play a role in the interconversion of three myoglobin forms, thereby influencing fillet coloration. The cytochrome c oxidase subunit II gene was a differentially expressed gene between red and chocolate ornamental shrimp [50].

Various studies have implicated ubiquitination as one of the regulatory mechanisms that determine meat quality in pork [71], lamb [72,73], and broiler chicken [74]. Ubiquitin carboxyl-terminal hydrolase-10 (*USP10*) is a member of the deubiquitinating enzyme family known as deubiquitinases, which include ubiquitin C-terminal hydroxylase-1 (*UCH-L1*) [75]. *USP10* and ubiquitin carboxyl-terminal hydrolase 47 (both on chromosome 6), respectively, explain 2.3% and ~2.0% of genetic variance associated with the yellowness phenotype in this study (Table 2). *UCH-L1* was implicated as influencing meat quality traits in pigs [76] and sheep [73]. *UCH-L1* reportedly regulates oxidative activity in skeletal muscle [77] and plays a role in myogenesis [78]. Polymorphism (A/G) in *USP10* causes a non-synonymous change in the amino acid from proline to leucine.

The peroxiredoxin family is a group of proteins capable of detoxifying peroxides and protecting cells against oxidation [79]. Peroxiredoxin-6 (*PRDX6*), on chromosome 4, explains 2.1% of genetic variance in fillet yellowness in the present fish population (Table 2). Proteome analysis of beef longissimus muscle revealed that peroxiredoxin-1 accounted for up to 70% of variances in color traits (L* a* b*) of muscle [80], and Wu et al. [79] identified peroxiredoxin as a possible marker for beef color. Peroxiredoxin-6 enzyme protects oxymyoglobin from peroxide attacks, thereby improving postmortem color stability [81]. Activator protein (AP-1) transcription factor on chromosome 6, which explains 1.7% of the genetic variance in fillet yellowness (Table 2), has been identified as a regulator of oxidative stress [82,83]. It protects the cell against reactive oxygen species. Other studies have identified a relationship between peroxiredoxin and meat quality or color traits in beef [84,85,86,87] and chevon [88]. Activation of the *AP-1* transcription factor induces the expression of many antioxidants, including peroxiredoxin and glutathione reductase [89,90]. It is possible that these genes (*PRDX6* and *AP-1*) function in the homeostatic regulation of the myoglobin redox state, protecting oxymyoglobin against oxidation and thereby enhancing the reddish coloration of the fillet.

Superoxide dismutase 2 (*SOD2*) encodes for muscle antioxidant enzyme. This enzyme reduces the damage caused by superoxide anion radicals [91]. Nohl et al. [92] identified superoxide dismutase as one of the agents protecting the mitochondria against lipid peroxidation and damage. Lipid oxidation and mitochondrial damage inhibit metmyoglobin reduction, and this causes muscle color deterioration [66]. *SOD2* on chromosome 8 explained 1.5% of the genetic variance in fillet whiteness (Table 3) in this study. A proteomics study on color stability in lamb identified *SOD2* as one of the proteins protecting the muscle against postmortem discoloration [93]. Superoxide dismutase was also a possible predictor of meat color stability in cattle [86] and chicken [94].

Sestrin-1 (*SESN-1*) on chromosome 8 explains ~1.4% of the genetic variance in fillet whiteness (Table 3). *SESN1* is known to confer resistance to oxidative stress through regenerating peroxiredoxins [95,96,97].

Hanan and Shaklai [98] reported a peroxidative interaction between myoglobin and myosin that regulates myoglobin homeostasis when attacked by a peroxide. In vitro oxidation of oxymyoglobin was significantly greater (*p* < 0.05) when in the presence of myosin compared to when myosin is absent in Tuna fish and Sardine [99]. Myosin X (*MYO10*) on chromosome 8 explains 1.3% of the genetic variance for fillet whiteness (Table 3). *MYO10* encodes for a myosin protein belonging to the myosin superfamily [100]. Myosin X may play a role in determining fillet color through its effect on oxymyoglobin oxidation.

Protein *PRRC2C* explains 2% of the genetic variability for fillet redness in this study (Table 2). Protein *PRRC2C* is required to efficiently form stress granules [101]. It is involved in the aggregation, arrangement, and bonding of proteins and RNA molecules to form a stress granule [101]. Stress granules are critical for facilitating responses against oxidative and cellular stress [102,103,104].

### 4.5. Genes Involved in Maintenance of Muscle Structural Integrity

The kelch protein 41b (*KLH41B),* collagen α-1(XXVIII) chain (*COL28A1*), myocilin *(MYOC),* F-actin-methionine sulfoxide oxidase (*MICAL2)*, and cathepsin K (*CTSK*) are genes that are found to affect fillet color in the study. They are known to be involved in the maintenance of muscle structural integrity. *KLH4LB* on chromosome 7 explains up to 3.4% of this study’s variance in the redness trait (Table 2). *KLH4LB* is involved in skeletal muscle cell differentiation, muscle fiber development, and sarcomere organization [105]. Functional studies of the role of the *KLH41B* gene in zebrafish revealed that its knockout resulted in myofibrillar disorganization and muscle weakness [106]. The relationship between structure and fillet color has been reported in the literature. Kiessling et al. [107] reported that fillets with higher L* (lightness) values were softer than those with low lightness values. Gagaoua et al. [108] identified protein biomarkers (α-actin and connectin) for beef color traits that are also structural proteins. The structural attributes of the muscle could influence the extent of light scattering for meat [109]. In their study on mice, Ramirez-Martinez et al. [110] showed that *KLH41B* maintains muscle function by preferentially helping stabilize nebulin, a protein needed to maintain muscle sarcomere integrity. They revealed that proteins involved in sarcomere organization and muscle contraction regulation were downregulated in *KLH41B* knockout mice. Loss of nebulin causes nemaline myopathy in humans, a condition associated with severe muscle weakness [111].

Collagen α-1(XXVIII) chain (*COL28A1*) harbors the SNP marker for muscle color in broiler chicken [112]. The same gene explained ~3.5% of the variance in fillet redness in this study (Table 2). Collagen is a connective tissue protein. The muscle extracellular matrix is mainly composed of collagen family proteins [113]. The relative amount and distribution of collagen fibers in the muscle can influence muscle quality [114].

Cathepsin K activity influenced skeletal muscle repair in mice [115]. Cathepsin K (*CTSK*) explains up to 2.8% of this study’s genetic variance in fillet redness (Table 2).

Myocilin *(MYOC)* encodes the protein myocilin, which is involved in regulating the actin cytoskeleton [116]. It explained 1.95% of the genetic variance in fillet yellowness in this study (Table 2).

F-actin-methionine sulfoxide oxidase (*MICAL2)* encodes methionine monooxygenase, which promotes depolymerization of F-actin by mediating the oxidation of residues on actin to form methionine-sulfoxide, resulting in actin filament disassembly and preventing repolymerization [117,118]. The gene is also involved in cytoskeleton organization [118]. It explains 1.9% of the genetic variance in fillet yellowness in this study (Table 2).

The cysteine-rich secretory protein LCCL domain-containing 2 (*CRISPLD2*) gene encodes a protein binding heparin and glycosaminoglycans and is involved in regulating the innate immune system [119]. It was downregulated as part of broiler chickens’ regulatory mechanisms for muscle pigmentation [120]. It explains 2.45% of the genetic variance in fillet yellowness in this study (Table 2).

### 4.6. SNP Variants Alter MicroRNA Binding Sites

MicroRNAs (miRNAs) are short non-coding RNAs between 20 and 24 nucleotides in length and can regulate gene expression post-transcriptionally by binding to the 3’UTR of its target mRNA [121,122]. This binding process can form the RNA-induced silencing complex (RISC) and subsequently repression of translation [123]. Mutation/polymorphism in miRNA and/or the target 3’UTR sequence have been associated with phenotypic variation in economically important traits. A G-to-A SNP substitution in the myostatin 3’UTR changes the miRNA target site and affects muscularity in sheep [124]. A C/G polymorphism in the precursor region of microRNA affects body weight, pelvis breadth, and chest depth in chickens [125,126].

The 3’UTR region of *ANKH* (ANKH inorganic pyrophosphate transport regulator), *RETRIG1* (reticulophagy regulator 1), and *HSPB1* (heat-shock protein, α-crystallin-related, 1) genes are target sites for omy-mir-1388-3p, omy-mir-219-5p, and omy-miR-724-5p microRNAs, respectively (Table 2 and Table 3). An A-to-T single nucleotide substitution at the target site of omy-mir-1388-3p causes a loss of its miRNA target site. Likewise, a C-to-T transition at the 3’UTR of *HSPB1* resulted in a loss of the target site for the omy-miR-724-5p miRNA. Single-nucleotide substitution at the target site of omy-mir-219-5p does not lead to loss of the target site.

Heat-shock proteins are common effectors of the cellular stress response. Thermal, environmental, or oxidative stress can trigger the transcription of genes encoding heat-shock proteins [127,128,129]. Protection of oxymyoglobin against oxidative stress is required to preserve bright-reddish meat coloration [66]. *HSPB1* encodes for a heat-shock protein that can protect against oxidative stress. Diet supplementation with antioxidant vitamins resulted in a significant drop in *HSPB1* expression in athletes after an exercise period compared with athletes fed an un-supplemented diet [130]. Over-expression of *HSPB1* has been shown to improve stress resistance (including oxidative stress) [130,131,132,133,134]. MicroRNA can repress the translation of its target mRNA. It is possible that the loss of the *HSPB1* target site facilitates the translation of the gene and induces resistance against oxidative stress.

Another gene that exhibits a loss of the miRNA target site under single-nucleotide polymorphism is the *ANKH* (ANKH inorganic pyrophosphate transport regulator). The gene encodes a protein that controls the extracellular level of pyrophosphate [135]. Inorganic pyrophosphate also plays an active role in oxidative stress resistance in several organisms [136,137,138].

## 5. Conclusions

We used weighted single-step GWAS to identify genetic variants associated with variability in fillet color traits in rainbow trout. Our result confirms that fillet color is a complex trait with no major gene but many SNP variants contributing to its regulation. We established that regulatory genes are involved in maintaining muscle structural integrity, carotenoid metabolism, or protection against myoglobin and lipid oxidation. An isoleucine-to-valine non-synonymous amino acid substitution mutation in β,β-carotene 15,15′-dioxygenase explained 2.2% of the phenotypic variance for yellowness, while SNP variants in retinol dehydrogenase-7 explained 2.9% of the variance in the muscle redness.

## Figures and Tables

**Table 1:** Descriptive statistics of the observed phenotypes.

Trait	N	Mean	SD	Min	Max	CV (%)	$\sigma_{a}^{2}$	$\sigma_{w}^{2}$	$\sigma_{e}^{2}$	h^2^ (SE)
Redness	878	1.98	1.06	−0.17	5.833	0.54	0.08	0.04	0.38	0.16 ± 0.06
Yellowness	878	4.41	1.31	−0.79	8.123	0.30	0.52	0.16	0.67	0.39 ± 0.07
Lightness	878	44.54	2.74	38.17	54.81	0.06	1.23	0.33	4.13	0.22 ± 0.07
Whiteness	878	44.3	2.64	38.11	54.22	0.06	1.13	0.31	3.92	0.21 ± 0.06

Where $\sigma_{a}^{2}$, $\sigma_{w}^{2}$, and $\sigma_{e}^{2}$ are the additive genetic variance, family variance, and residual variance, respectively, and h^2^ is the heritability estimate.

**Table 2:** Selected SNP markers within 50 SNPs’ genomic sliding windows, explaining at least 1% of the additive genetic variance for fillet redness and yellowness traits.

Redness					
Chr	POS	%Var	Gene ID	Gene Annotation	Region/Effect
7	10,996,914	2.43	LOC110527401	Radixin	CDS/syn
7	11,138,396	2.49	LOC110527405	Calsequestrin-2	CDS/Syn
7	11,312,252	2.73	LOC110527407	Zinc finger protein Dzip1	CDS/syn
7	11,399,310	3.45	LOC110527414	Kelch protein 41b	CDS/syn
7	11,402,881	3.47	LOC110527413	Collagen α-1(XXVIII) chain	3’UTR
7	11,438,574	3.29	LOC100136600	ATP synthase subunit β, mitochondrial	CDS/syn
7	11,444,638	3.02	LOC110527417	Retinol dehydrogenase 7	CDS/syn
7	11,459,018	2.98	abcb11	Bile salt export pump	CDS/syn
7	11,477,215	2.88	LOC100136260	Cathepsin K	CDS/syn
9	52,063,734	2.27	LOC110532529	Tyrosine-protein phosphatase non-receptor type 1	3’UTR
9	52,106,708	2.26	LOC110532530	Ubiquitin-conjugating enzyme E2 variant 1	3’UTR
9	52,291,239	2.28	LOC110532539	Partner of Y14 and mago A	CDS/syn
12	53,800,425	1.1	hspb1	Heat-shock protein, α-crystallin-related-1	3’UTR/miRNA target
**Yellowness**					
4	22,957,625	2.09	prdx6	Peroxiredoxin 6	CDS/syn
4	22,973,619	2.11	plpp6	Phospholipid phosphatase 6	5’UTR
4	23,074,540	2	LOC110521622	Protein PRRC2C	3’UTR
4	23,103,208	1.92	vamp4	Vesicle-associated membrane protein 4	3’UTR
4	23,115,313	1.95	LOC110521624	Myocilin	CDS/Syn
6	61,578,946	1.9	LOC110526379	F-actin-methionine Sulfoxide oxidase MICAL2	5’UTR
6	61,592,297	1.99	LOC110526380	Ubiquitin carboxyl-terminal hydrolase 47	CDS/syn
6	61,666,093	2.25	LOC110526946	β,β-carotene 15,15′-dioxygenase-l	CDS/Non-syn
6	61,805,211	2.11	LOC110526388	Nuclear factor of activated T-cells 5	CDS/syn
6	61,837,913	2.39	LOC110526389	Lysine-tRNA ligase	3’UTR
6	61,847,413	2.38	LOC110526390	60S ribosomal protein L13	CDS/syn
6	61,998,041	2.36	LOC110526393	Cytochrome b5	3’UTR
6	62,768,347	2.45	LOC110526402	Cysteine-rich Secretory protein LCCL domain-containing 2	3’UTR
6	62,812,905	2.32	LOC110526403	Ubiquitin carboxyl-terminal hydrolase 10	CDS/Non-syn
6	62,896,859	2.24	LOC110526405	AP-1 complex subunit γ-1	3’UTR
6	62,961,238	2.26	LOC110526408	Myotubularin-related Protein 10	3’UTR
6	63,056,828	2.39	LOC100136691	Cyclin B2	CDS/syn

Chr = chromosome, POS = SNP position %Var = % variance explained, Syn = synonymous amino acid substitution, Non-Syn = non-synonymous amino acid substitution. Color intensities (green, yellow, and red) reflect changes in additive genetic variance explained by the SNP genomic sliding window for the fillet trait. A color gradient indicates differences in additive genetic variance explained by windows containing the representative SNP marker (green is the highest and red is the lowest).

**Table 3:** Selected SNP markers within 50 SNPs’ genomic sliding windows, explaining at least 1% of the additive genetic variance for the fillet whiteness trait.

		Whiteness			
Chr	POS	%Var	Gene ID	Gene Annotation	Region/Effect
8	34,097,292	1.17	LOC110529884	Peptidyl-prolyl cis-trans isomerase FKBP1B	CDS/Syn
8	34,136,112	1.29	mut	Methylmalonyl-CoA mutase	3’UTR
8	34,495,040	1.49	sod2	Superoxide dismutase 2	3’UTR
8	34,936,875	1.57	LOC110529892	cGMP-dependent protein kinase 1	3’UTR
8	36,538,411	1.42	LOC110529899	SAM and SH3 domain-containing protein 1	3’UTR
8	37,290,793	1.54	LOC110529911	Sialomucin core protein 24	3’UTR
8	37,412,186	1.38	LOC110529910	Sestrin-1	3’UTR
8	37,829,107	1.38	ostm1	Osteopetrosis-associated transmembrane protein 1	3’UTR
8	38,254,068	1.3	LOC110529920	Poly(U)-binding-splicing factor PUF60	CDS/Syn
8	39,295,098	1.37	ankh	ANKH inorganic pyrophosphate transport regulator	3UTR/miRNA target
8	40,954,559	1.3	myo10	Myosin X	3’UTR
8	40,978,990	1.36	znf622	Zinc finger protein 622	3’UTR
8	41,002,542	1.26	retreg1	Reticulophagy regulator 1	3’UTR/miRNA target
19	41,952,271	1.18	LOC110497982	Uncharacterized protein C15orf52	3’UTR
27	1,675,710	1.19	LOC110507317	Protein IWS1 homolog	3’UTR
27	3,976,684	1.18	LOC110507360	Serine/threonine-protein phosphatase 2A 65 kDa regulatory subunit A β isoform	CDS/Syn

Chr = chromosome, POS = SNP position, %Var = % variance explained, Syn = synonymous amino acid substitution. Color intensities (green, yellow, and red) reflect changes in additive genetic variance explained by the SNP genomic sliding window for the fillet trait. A color gradient indicates differences in additive genetic variance explained by windows containing the representative SNP marker (green is the highest and red is the lowest).

## Data Availability

All datasets generated for this study are included in the manuscript and/or the Supplementary Files. The genotypes (ped and map files) and phenotypes are available in our previous publication [9].

## Supplementary Materials

The following supporting information can be downloaded at: https://www.mdpi.com/article/10.3390/genes13081331/s1, Table S1: SNPs and genes identified in this study explaining at least 1% of the genetic variation in fillet redness, yellowness, and whiteness.

## Abbreviations

GWA: genome-wide association; HWE: Hardy–Weinberg equilibrium; LD: linkage disequilibrium; MAF: minor allele frequency; NCCCWA: USDA National Center of Cool- and Cold-Water Aquaculture; QC: quality control; QTL: quantitative trait loci; SNP: single-nucleotide polymorphism; UTR: untranslated region; WssGBLUP: weighted single-step GBLUP; YC: year class, L, a, b, w.
